# Nasopharyngeal carriage rate of *Streptococcus pneumoniae *in Ugandan children with sickle cell disease

**DOI:** 10.1186/1756-0500-5-28

**Published:** 2012-01-13

**Authors:** David P Kateete, Henry Kajumbula, Deogratias H Kaddu-Mulindwa, Augustine K Ssevviri

**Affiliations:** 1 Department of Medical Microbiology, School of Biomedical Sciences, Makerere University College of Health Sciences, Box 7072 Kampala, Uganda

## Abstract

**Background:**

Nasopharyngeal carriage of *Streptococcus pneumoniae *is a determinant for invasive pneumococcal disease, which often complicates homozygous sickle cell disease. Here, we determined the nasopharyngeal carriage rate of *S. pneumoniae *in Ugandan children with homozygous sickle cell disease, who attended the outpatient Sickle Cell Clinic at Mulago National Referral hospital in Kampala, Uganda.

**Results:**

*S. pneumoniae *occurred in 27 of the 81 children with homozygous sickle cell disease (giving a carriage rate of 33%, 27/81). Twenty three children were previously hospitalized of whom *S. pneumoniae *occurred in only two (9%, 2/23), while among the 58 who were not previously hospitalized it occurred in 25 (43%, 25/58, χ^2 ^= 8.8, *p *= 0.003), meaning there is an association between high carriage rate and no hospitalization. Two children previously immunized with the pneumococcal conjugate vaccine did not carry the organism. Prior antimicrobial usage was reported in 53 children (65%, 53/81). There was high resistance of pneumococci to penicillin (100%, 27/27) and trimethoprime-sulfamethoxazole (97%, 26/27), but low resistance to other antimicrobials. Of the 70 children without sickle cell disease, *S. pneumoniae *occurred in 38 (54%, 38/70) of whom 43 were males and 27 females (53% males, 23/43, and 56% females, 15/27).

**Conclusion:**

Nasopharyngeal carriage of penicillin resistant pneumococci in Ugandan children with homozygous sickle cell disease is high. While nasopharyngeal carriage of *S. pneumoniae *is a determinant for invasive pneumococcal disease, pneumococcal bacteremia is reportedly low in Ugandan children with sickle cell disease. Studies on the contribution of high carriage rates to invasive pneumococcal disease in these children will be helpful. This is the first report on pneumococcal carriage rate in Ugandan children with sickle cell disease.

## Background

Sickle cell anemia, also known as homozygous sickle cell disease (genotype *β^s^β^s ^*[1]) is prevalent in Uganda [2]. Invasive pneumococcal disease is one of the leading complications of homozygous sickle cell disease (HbSS) [3]. Patients with HbSS are more prone to pneumococcal meningitis [4,5] and to pneumococcal septicemia [6,7]. Globally, the incidence of invasive pneumococcal disease in children with HbSS is 30- to 600-fold higher than in individuals of comparable age and race without HbSS; pneumococcal septicemia and meningitis are important causes of death in HbSS patients, with case/fatality rates of 15 to 35% [4,8-11].

There are varying reports of invasive pneumococcal disease in sub-Saharan African children with HbSS; in the Democratic Republic of Congo, *S. pneumoniae *was the most frequent isolate from HbSS patients with bacteremia [2,12], and the organism was recovered from blood cultures of all the eight HbSS-patients with pneumonia in Northern Nigeria [13]. However, three other Nigerian studies [14-16] reported low prevalence of pneumococcal bacteremia in patients with HbSS. Further, *S. pneumoniae *bacteremia is reportedly very low in Ugandan children with HbSS [17].

Nasopharyngeal carriage of *S. pneumoniae *is the determinant for invasive pneumococcal disease particularly in industrialized settings [18,19]; therefore, investigation of pneumococcal colonization is important particularly in high risk groups such as HbSS patients. While pneumococcal bacteremia in Ugandan children with HbSS is low [17], pneumococcal carriage rates in these children have not been determined, yet carriage rates for healthy children are available (62% prevalence [20]). Here we aimed to determine the nasopharyngeal carriage rate of *S. pneumoniae *in Ugandan children with HbSS attending the Sickle Cell Clinic at Mulago National Referral Hospital in Kampala, Uganda.

## Methods

### Patient description and sampling

This study took place over a 6 month period from December 2001 to May 2002. Eighty one children (51 males and 30 females, aged 8 months to 6 years, ascertained from patient records) with severe HbSS, attending the Sickle Cell Clinic at Mulago hospital in Kampala, Uganda, were recruited. Of these, 23 were previously admitted for treatment of low haemoglobin levels. All the children frequently visited the sickle cell clinic, which provides care to children confirmed with sickle cell anemia in Uganda (see references [2] and [17]). The youngest child was 8 months while the oldest was 6 years; the median age was 2.8 years. All the children had symptoms of severe HbSS disease based on clinical reports and symptoms. Records indicated that the children were on programmed routine visits to the clinic, although some reported whenever complications developed. The diagnosis of HbSS was based on an FSA_2 _pattern upon haemoglobin electrophoresis and characteristic haematology; haemoglobin electrophoresis testing was performed earlier on samples from these children and detected homozygous sickle cell disease (HbSS).

Parents or guardians signed an informed consent form and were interviewed on antimicrobial usage during the preceding one month and on the history of pneumococcal immunization (to determine their effects on recovery of pneumococci). The data on antimicrobial usage was verified by cross-checking with the information on prescription forms presented by the attendants. Nasopharyngeal specimens (one sample per child) were obtained by a paediatrician on duty using pre-packed sterile disposable calcium alginate fiber tipped-aluminum applicator swab. Children above 6 years were excluded (because they are less colonized by pneumococci [21]), as well as those who presented with severe ailments particularly pneumonia, and those who lacked consent from parents/guardians. Seventy children (2-6 years) with no history of HbSS from an outpatient ward were also sampled (controls) to compare the pneumococcal carriage rates. Symptoms of HbSS were missing in control subjects and they were considered not to have HbSS; however, they presented with fever and diarrhea, for which they sought care. For most children in Uganda, HbSS symptoms usually manifest within 6 months to 1 year after birth (unpublished observations); hence we excluded children less than 1 year among controls to minimize including those with HbSS disease.

### Culture and identification of *S. Pneumoniae*

Nasopharyngeal specimens were immediately transported in Stuart's transport medium to the laboratory and cultured according to standard microbiological procedures. Briefly, the specimens were inoculated onto 5% rabbit blood agar plates and incubated for 24-48 h at 35-37°C under 5% CO_2_. *S. pneumoniae *was identified based on colony morphology, α-hemolysis and Gram staining and confirmed based on optochin sensitivity and bile solubility. Antimicrobial susceptibility was performed with bacterial suspensions of turbidity equivalent to McFarland 0.5 with the following disks (Oxoid, UK): oxacillin, 1 μg; erythromycin, 25 μg; ceftriaxone, 30 μg; chloramphenicol, 30 μg; trimethoprime-sulfamethoxazole, 23-75 μg; rifampicin, 5 μg; and perfloxacin, 17 μg. Interpretation for sensitivity, intermediate or resistance was based on guidelines from the Clinical and Laboratory Standards Institute (CLSI). Controls included *Staphylococcus aureus *ATCC 25923 and *Escherichia coli *ATCC 29522.

Ethical approval was obtained from the Mulago Hospital Research and Ethics Committee, and the Faculty of Medicine Research and Ethics Review Board.

## Results and discussion

*S. pneumoniae *occurred in 27 of the 81 children with HbSS (giving a nasopharyngeal carriage rate of 33%, 27/81). Thus, in the current study a much higher *S. pneumoniae *carriage rate was found than in a previous report from the USA in which prevalence was reported to be 13% [3]. However, for healthy Ugandan children without HbSS, Joloba et al, found a significantly higher nasopharyngeal carriage rate (62%) [20]. Of the 23 previously hospitalized children, pneumococci occurred in two (2/23, 9%). Fifty eight children had not been hospitalized before, of whom pneumococci occurred in 25, meaning there is an association between high carriage rate and no hospitalization (25/58, 43%, χ2 = 8.8, *p *= 0.003). There was no major difference in carriage rates between males and females (i.e., 35%, 18/33 males vs. 30%, 9/23 females). Of the 70 children without HbSS, pneumococci occurred in 38 (54%, 38/70) of whom 23 were males (23/38, 61%) and 15 females (15/38, 39%). Two children with HbSS had been immunized with a conjugate pneumococcal vaccine and did not carry the organism; pneumococcal immunization was not reported among children without HbSS. Fifty three children with HbSS reported antimicrobial usage (53/81, 65%, Table 1) while it was reported by 43 children without HbSS (43/70, 61%). Antimicrobials commonly used included penicillins, sulphomexazole-trimethoprim, chloramphenicol and cephalexin (Table 1).

**Table 1:** Antimicrobial usage in Ugandan children with HbSS

Antimicrobial		None	SXT	Pen	Amo/Clox	Aug	Amo/SXT	Amp	Chl	Cep
*S. pneumoniae *carriers	**27**	9	7	**5**	1	1	2	1	1	-

Non carriers	**54**	19	12	**4**	4	3	4	5	2	1

**TOTAL**	**81**	28	19	**9**	5	4	6	6	3	1

SXT, trimethoprime-sulfamethoxazole; Pen, penicillin; Amo, amoxicillin; Clox, cloxacillin; Aug, Augmentin; Amo, amoxicillin; Amp, ampicillin; Chl, chloramphenicol; Cep, cephalexin; -, Not detected

All isolates from children with HbSS were resistant to penicillin (27/27, 100%) while those from children without HbSS were intermediate (38/38, 100%). Resistance to sulphomexazole-trimethoprim was also high for both categories. However, the isolates from both groups were sensitive to the other commonly used antimicrobials (Table 2). These findings are similar to those of Joloba et al, who found high rates of penicillin and sulphomexazole-trimethoprim resistant *S. pneumoniae *[20]. However in this study, the isolates from children with HbSS were fully resistant to penicillin while those in the previous study were intermediate. The lower carriage rate and increased antimicrobial resistance to penicillin in the current study may be attributed to the common usage of penicillin in children with HbSS [20]. Nevertheless, there is no report on penicillin prophylaxis among sickle cell children in Uganda but the drug is widely prescribed [2] and can be purchased over the counter without prescription. Further, while penicillin prophylaxis in Uganda is recommended for children with HbSS, it is not followed in principle since the drug is not free and some parents/guardians cannot afford it. Usually parents with the means to do so can easily obtain the drug over the counter. However, when the children are unwell the drug will almost certainly be taken, meaning that use of penicillin in this population is generally symptomatic rather than prophylactic. High carriage rates of antimicrobial resistant strains were also found in Zambia (*Pneumococcus *was reported in 71.9% of the children [22]); however, 12.7% of the strains were resistant to penicillin [22]. The reason for the difference in resistance rates between Uganda and Zambia is likely due to law enforcement practices on antimicrobial usage.

**Table 2:** Antimicrobial susceptibility patterns of pneumococci from Ugandan children with HbSS

	HbSS			No HbSS		
**Antimicrobial**	**Susceptible (%)**	**Intermediate (%)**	**Resistant (%)**	**Susceptible (%)**	**Intermediate (%)**	**Resistant (%)**

Penicillin	-	-	**27 (100)**	-	**38 (100)**	-

SXT	-	1 (3)	**26 (97)**	-	**5 (7)**	**18 (26)**

Chloramphenicol	26 (97)	-	1 (3)	16 (23)	2 (3)	5 (7)

Erythromycin	26 (97)	-	1 (3)	19 (27)	2 (3)	2 (3)

Rifampin	27 (100)	-	-	23 (33)	-	-

Cefriaxone	27 (100)	-	-	23 (33)	-	-

Perfloxacin	17 (100)	-	-	23 (33)	-	-

-, Not detected

Antimicrobial usage during the previous month did not significantly affect nasopharyngeal carriage rates (*p *= 0.93). However in previous studies, prior antimicrobial usage reduced the nasopharyngeal carriage of *S. pneumoniae *[23]. Of the 27 HbSS children from whom *S. pneumoniae *was recovered, nine had not used antimicrobials; seven had used sulphomexazole-trimethoprim; five penicillin; one amoxicillin-cloxacillin; one Augmentin; two amoxicillin + sulphomexazole-trimethoprim; one ampicillin; one chloramphenicol and none used cephalexin. Of the 54 HbSS children from whom *S. pneumoniae *was not recovered, 19 had not used antimicrobials; 12 had used sulphomexazole-trimethoprim; three penicillin; four amoxicillin-cloxacillin; three Augmentin; four amoxicillin + sulphomexazole-trimethoprim; five ampicillin; two chloramphenicol and one cephalexin. Of the 70 children without HbSS, 27 had not used any antimicrobial; 27 had taken sulphomexazole-trimethoprim; 12 penicillin; three chloramphenicol; and one amoxicillin + sulphomexazole-trimethoprim.

Despite the high nasopharyngeal carriage (which is a determinant for invasive pneumococci [18]), there is a puzzling discrepancy of high carriage rates with low rates of invasive pneumococcal disease in sub-Saharan Africa (see references [1,17]). Kizito et al [17] reported a significantly low rate of pneumococcal bacteremia in Ugandan children with sickle cell disease (6%, 3/47) [17], and other studies have indicated low pneumococcal bacteremia in Nigerian children [1,14-16]. These could be a consequence of uncontrolled antimicrobial usage particularly in the urban populations in those countries. However, the high rates of pneumococcal bacteremia in children with/without sickle cell disease in neighboring Kenya [6], the Gambia [24], Ghana [25], and Mozambique [26] cast doubt on previous reports with low rates of invasive pneumococcal disease. Since laboratory facilities in sub-Saharan Africa are generally not well established [6,27], surveillance can be challenging particularly for fastidious organisms such as the pneumococcus. Moreover, certain invasive pneumococcal serotypes are difficult to detect in the nasopharynx. Further, since the largest population based retrospective study of bacteremia in Kenyan children with HbSS revealed high incidence of invasive pneumococci [6], contrary to current reports [1,17], invasive pneumococcal disease may not be uncommon in sub-Saharan African children with HbSS.

One major limitation in this study was failure to determine the prevailing serotypes among HbSS patients, which we presume would most likely be similar to those described by Joloba et al (i.e., serogroups 6, 9, 14, 19, and 23) [20]. Further, due to limited resources, we were unable to extend the study beyond 6 months, and we did not confirm control subjects for the absence of HbSS disease but relied on absence of HbSS symptoms. This being the first report of nasopharyngeal carriage rate of pneumococci from Ugandan children with sickle cell disease, we hope that future studies will put these in consideration.

## Conclusions

A high carriage rate of penicillin resistant *S. pneumoniae *has been found in Ugandan children with HbSS. This rate is similar to or higher than those in settings where nasopharyngeal carriage is the determinant for invasive pneumococcal disease in HbSS patients [3]. A puzzling finding in previous studies is the low level of pneumococcal bacteremia in Ugandan children with HbSS [17]. Accordingly, the usefulness of pneumococcal prophylaxis in Ugandan children with HbSS has been debated [17]. Since *pneumococci *are leading causes of childhood and adult bacteremia/mortality in sub-Saharan Africa [26,28-30], further studies are necessary to resolve the discrepancy between high nasopharyngeal carriage rates of pneumococci and invasiveness.

## Abbreviations

*β^s^β^s^*: Genotype for homozygous sickle cell disease encoding haemoglobin S (Hbs an abnormal version of beta-globin); HbSS: Homozygous sickle cell disease; MakCHS: Makerere University College of Health Sciences.

## Competing interests

The authors declare that they have no competing interests.

## Authors' contributions

AKS conceived, planned and conducted the study; DPK and AKS wrote the manuscript; HK and DHK supervised the study and helped with data analysis. All authors read and approved the final version of the manuscript.
